# Evaluation of Ligand-Inducible Expression Systems for Conditional Neuronal Manipulations of Sleep in *Drosophila*

**DOI:** 10.1534/g3.116.034132

**Published:** 2016-08-23

**Authors:** Qiuling Li, Nicholas Stavropoulos

**Affiliations:** Department of Neuroscience and Physiology, Neuroscience Institute, New York University School of Medicine, New York 10016

**Keywords:** Geneswitch, RU486, Q-system, insomniac, Cul3, FlyBook

## Abstract

*Drosophila melanogaster* is a powerful model organism for dissecting the molecular mechanisms that regulate sleep, and numerous studies in the fly have identified genes that impact sleep–wake cycles. Conditional genetic analysis is essential to distinguish the mechanisms by which these genes impact sleep: some genes might exert their effects developmentally, for instance by directing the assembly of neuronal circuits that regulate sleep; other genes may regulate sleep in adulthood; and yet other genes might influence sleep by both developmental and adult mechanisms. Here we have assessed two ligand-inducible expression systems, Geneswitch and the Q-system, for conditional and neuronally restricted manipulations of sleep in *Drosophila*. While adult-specific induction of a neuronally expressed Geneswitch transgene (*elav-GS*) is compatible with studies of sleep as shown previously, developmental induction of *elav-GS* strongly and nonspecifically perturbs sleep in adults. The alterations of sleep in *elav-GS* animals occur at low doses of Geneswitch agonist and in the presence of transgenes unrelated to sleep, such as *UAS-CD8-GFP*. Furthermore, developmental *elav-GS* induction is toxic and reduces brood size, indicating multiple adverse effects of neuronal Geneswitch activation. In contrast, the transgenes and ligand of the Q-system do not significantly impact sleep–wake cycles when used for constitutive, developmental, or adult-specific neuronal induction. The nonspecific effects of developmental *elav-GS* activation on sleep indicate that such manipulations require cautious interpretation, and suggest that the Q-system or other strategies may be more suitable for conditional genetic analysis of sleep and other behaviors in *Drosophila*.

The establishment of *Drosophila* as a model organism for studies of sleep (Hendricks *et al.* 2000; Shaw *et al.* 2000) has facilitated unbiased and candidate-based screens for genes that impact sleep–wake cycles (*e.g.*, Cirelli *et al.* 2005; Kume *et al.* 2005; Koh *et al.* 2008). While these studies have revealed an increasing number of genes that influence sleep, the underlying mechanisms are in most cases poorly defined. One critical aspect of elucidating these mechanisms is defining the temporal window in which genes function in relation to sleep. Some genes might function principally in a developmental manner, for example by contributing to the assembly of neuronal circuits relevant to sleep, while other genes might function specifically in adulthood and regulate sleep in a sustained or dynamic manner. Given that most *Drosophila* genes are expressed at multiple stages of the life cycle (Graveley *et al.* 2011) and that many genes have pleiotropic functions, individual genes may impact sleep by both developmental and adult mechanisms. Temporally restricted genetic manipulations are therefore essential to distinguish among these mechanisms and to shape hypotheses for how various genes exert their effects on sleep.

Strategies for temporally restricted genetic manipulations in *Drosophila* use temperature, chemical ligands, and light as conditional triggers. Temperature-regulated systems include heat-inducible promoters fused directly to heterologous genes (Lis *et al.* 1983) or to the Gal4 activator (Brand *et al.* 1994), and the Gal80^ts^ system in which a temperature-sensitive form of the Gal80 suppressor restricts Gal4 activity (McGuire *et al.* 2003). Ligand-inducible systems include tetracycline-dependent activators (Bello *et al.* 1998; Bieschke *et al.* 1998; Stebbins *et al.* 2001), steroid-activated forms of Gal4 (Osterwalder *et al.* 2001; Roman *et al.* 2001), and the Q-system derived from *Neurospora* (Potter *et al.* 2010; Riabinina *et al.* 2015). More recently, photosensitive transcriptional activators have been described (Chan *et al.* 2015). Of these strategies, the steroid-inducible Geneswitch system (Osterwalder *et al.* 2001; Roman *et al.* 2001) has been the most frequently employed to study sleep and has been used in nearly two dozen such studies to date (Yuan *et al.* 2006; Joiner *et al.* 2006; Seugnet *et al.* 2008, 2011; Bushey *et al.* 2009; Donlea *et al.* 2009; Wu *et al.* 2009; Kuo *et al.* 2010, 2012; Crocker *et al.* 2010; Ishimoto and Kitamoto 2010; Pfeiffenberger and Allada 2012; Erion *et al.* 2012; Ueno *et al.* 2012; Vanderheyden *et al.* 2013; Tulina *et al.* 2014; Liu *et al.* 2014; Kayser *et al.* 2014; Oh *et al.* 2014; Chen *et al.* 2015; Tabuchi *et al.* 2015; Dissel *et al.* 2015; Afonso *et al.* 2015).

Geneswitch is a tripartite fusion protein containing the Gal4 DNA-binding domain, the progesterone receptor ligand-binding domain, and the p65 transcriptional activation domain (Burcin *et al.* 1999). In the presence of RU486, a progesterone receptor agonist, Geneswitch induces the transcription of genes located downstream of the upstream activating sequence (UAS) element bound by Gal4 (Burcin *et al.* 1999). In *Drosophila*, spatial restriction of Geneswitch activity is conferred by tissue-specific regulatory elements, while temporal control is achieved by delivery of RU486 in a restricted manner during development or adulthood (Osterwalder *et al.* 2001; Roman *et al.* 2001).

Nearly all of the reported uses of Geneswitch to manipulate sleep in *Drosophila* have utilized adult-specific induction, achieved by feeding RU486-containing food to adult flies (Yuan *et al.* 2006; Joiner *et al.* 2006; Seugnet *et al.* 2008, 2011; Bushey *et al.* 2009; Donlea *et al.* 2009; Wu *et al.* 2009; Kuo *et al.* 2010, 2012; Crocker *et al.* 2010; Ishimoto and Kitamoto 2010; Pfeiffenberger and Allada 2012; Erion *et al.* 2012; Ueno *et al.* 2012; Vanderheyden *et al.* 2013; Tulina *et al.* 2014; Liu *et al.* 2014; Kayser *et al.* 2014; Oh *et al.* 2014; Chen *et al.* 2015; Tabuchi *et al.* 2015; Dissel *et al.* 2015; Afonso *et al.* 2015). RU486 is well tolerated in adults with no detectable toxic effects at high concentrations (500 µM) (Osterwalder *et al.* 2001; Roman *et al.* 2001), and studies of sleep have typically used concentrations at or below this threshold to activate Geneswitch transgenes expressed neuronally (*elav-GS*) (*e.g.*, Joiner *et al.* 2006; Seugnet *et al.* 2008; Bushey *et al.* 2009), in mushroom bodies (*MB-GS*) (Yuan *et al.* 2006; Joiner *et al.* 2006; Wu *et al.* 2009; Ishimoto and Kitamoto 2010), in fat bodies (*S_1_106-GS*) (Kuo *et al.* 2010), and ubiquitously (*da-GS*) (Tabuchi *et al.* 2015; Dissel *et al.* 2015). In adult animals, Geneswitch and RU486 do not alter sleep in the absence of effector transgenes (*e.g.*, Wu *et al.* 2009; Afonso *et al.* 2015), permitting use of the Geneswitch system for various adult-specific manipulations of sleep.

In contrast to the many studies of sleep that have used adult-specific Geneswitch induction, few have used constitutive or developmental-specific induction (Kuo *et al.* 2010; Pfeiffenberger and Allada 2012). One relevant concern is the developmental toxicity of RU486 at concentrations lower than those tolerated in adulthood (Osterwalder *et al.* 2001; van Swinderen 2007; Shen *et al.* 2009; Landis *et al.* 2015). In the absence of Geneswitch transgenes, high concentrations of RU486 are intrinsically toxic to early *Drosophila* development, as indicated by reduced numbers of larvae arising from parents fed 233 µM RU486 (Osterwalder *et al.* 2001). The toxicity of RU486 appears to be increased in the presence of *elav-GS* and effector transgenes, as suggested by developmental lethality from exposure to RU486 concentrations greater than 25 µM (van Swinderen 2007). While the threshold for developmental toxicity of Geneswitch is not well defined, low concentrations of RU486 (11.6 µM = 5 µg/ml) were found to be permissive for larval development in the context of ∼200 different Geneswitch drivers (Nicholson *et al.* 2008). In addition to its acute effects on development, early Geneswitch activation can have adverse consequences later in life, as indicated by reduced lifespan of *elav-GS* animals exposed to RU486 during development (Shen *et al.* 2009).

The impact of developmental Geneswitch activation on sleep and other adult behaviors has not been assessed comprehensively. Constitutive activation of the *elav-GS* and *S_1_106-GS* drivers was reported in a study of the immune response and sleep (Kuo *et al.* 2010), though sleep was assessed in a narrow window postinjury and effects on total daily sleep were not determined. A second study reported developmental-specific *elav-GS* induction, by setting crosses on RU486-containing food and moving adults to food lacking RU486 (Pfeiffenberger and Allada 2012). While developmental toxicity was not addressed, sleep was reported to be near normal in *elav-GS* animals exposed developmentally to 50 µM RU486 (Pfeiffenberger and Allada 2012). Additional studies are required to assess the general utility of developmental Geneswitch activation, and to compare this strategy to other conditional manipulations of sleep.

The Q-system is a more recently developed ligand-inducible system that utilizes components of the *Neurospora crassa* quinic acid gene cluster (Potter *et al.* 2010): the QF transcriptional activator, the QS suppressor that binds and inhibits QF in a quinic acid-dependent manner, and the QUAS regulatory element bound by QF. A refinement of the Q-system is the hybrid Gal4QF activator, in which the Gal4 DNA-binding domain replaces that of QF, enabling activation of UAS transgenes while preserving suppression by QS and derepression by quinic acid (Riabinina *et al.* 2015). Quinic acid has no obvious adult toxicity (Potter *et al.* 2010) and panneuronal QF expression does not significantly alter circadian rhythms or sleep (Riabinina *et al.* 2015). However, the effects of quinic acid exposure on sleep have not been determined in animals lacking or expressing Gal4QF and QS, components necessary for conditional activation of UAS transgenes. Thus, whether the Q-system is compatible with developmental and adult manipulations of sleep is not yet known.

Here we have evaluated Geneswitch and the Q-system for conditional, neuronally restricted manipulations of sleep. While adult-specific induction of the panneuronal *elav-GS* driver is compatible with assessing sleep as reported in earlier studies, developmental-specific or constitutive induction of *elav-GS* causes developmental defects and nonspecific reductions of sleep in adulthood. In contrast, the constituent transgenes and inducing ligand of the Q-system do not alter sleep in developmental-specific, adult-specific, and constitutive neuronal manipulations. Our findings indicate that nonspecific perturbations of sleep caused by developmental *elav-GS* induction preclude such manipulations for studies of sleep, and that the Q-system may have broader utility for systematically defining the temporal windows in which genes impact sleep and other behaviors in *Drosophila*.

## Materials and Methods

### Stocks and transgenes

*elav-GS* (Osterwalder *et al.* 2001), *UAS-dcr2* (Bloomington #24651; Dietzl *et al.* 2007), *UAS-CD8-GFP* (Bloomington #5137; Lee and Luo 1999), *tub-QS* (Potter *et al.* 2010), *nsyb-Gal4QF* (Riabinina *et al.* 2015), and *UAS-inc-RNAi* (VDRC 18225; Dietzl *et al.* 2007) were described previously. *elav-GS* in the *iso31* background (Ryder *et al.* 2004) was described previously (Crocker and Sehgal 2008). A third chromosome insertion of *nsyb-Gal4QF* and two different second chromosome *tub-QS* insertions were each backcrossed eight generations to the *iso31* background. After backcrossing, both *tub-QS* insertions were separately combined with *nsyb-Gal4QF* to yield *tub-QS*; *nsyb-Gal4QF* stocks. The *elav-GS*, *UAS-dcr2* stock was obtained by meiotic recombination; two independently derived recombinants were verified by PCR and behaved similarly. *UAS-CD8-GFP* was used in its existing genetic background. All experiments utilized male animals bearing one copy of indicated transgenes.

### Drosophila culture and conditional induction of Geneswitch and the Q-system

For all experiments, crosses were performed with five virgin females and three males and supplemented with yeast in standard fly vials (28.5 mm outer diameter × 95 mm height) and cultured at 25° in alternating 12 hr cycles of light and darkness (LD). Flies were cultured on food containing the following ingredients: 1800 g cornmeal (Labscientific, FLY-8010-20), 1800 ml molasses (Labscientific, FLY-8008-16), 744 g yeast (Labscientific, FLY-8040-20F), 266 g agar (Mooragar, 41084), 56 g Tegosept (Sigma, H3647), 560 ml alcohol (Fisher, A962P4), 190 ml propionic acid (Fisher, A258500), and 47 l of water. To prepare food for conditional induction experiments, solid food was melted in a microwave oven and cooled before addition of RU486, quinic acid, or appropriate vehicle.

For developmental (**ON→OFF**) Geneswitch induction, crosses were set on food containing RU486 (Sigma, M8046) at 11.6, 50, or 500 μM. One- to four-day-old young adults eclosing from these cultures were moved to vehicle-containing food (ethanol at a maximum 0.43% concentration) in *Drosophila* Activity Monitoring (DAM) tubes (Trikinetics) for behavioral assay. For constitutive (**ON**) Geneswitch induction, crosses were set on 11.6 μM RU486, and young adult progeny were moved to DAM tubes containing 500 µM RU486. For adult-specific Geneswitch induction (**OFF→ON**), crosses were set on food containing vehicle, and young adult progeny were moved to DAM tubes containing 500 µM RU486. For the negative control condition (**OFF**), crosses were set, and young adults were assayed, on vehicle-containing food.

Q-system induction was performed similarly, using food containing quinic acid or vehicle as dictated by the four different induction regimens. Quinic acid (Sigma, 138622) was freshly prepared by dissolving 1 g of quinic acid in 3 ml of water, and 330 μl of this solution was added per 10 ml of fly food. An equal volume of water was used for vehicle controls.

For assessing developmental toxicity of Geneswitch, vials were photographed and total numbers of pupae above the food surface were counted for all genotypes 10 or 11 d after setting crosses, as noted in the figure legends.

### Sleep analysis

For measurements of sleep, 1–4-d-old male animals eclosing from LD-entrained cultures raised at 25° were loaded into glass DAM tubes (5 mm diameter × 65 mm length) containing food, and RU486, quinic acid, or appropriate vehicle as dictated by induction conditions. Animals were assayed for 7 d at 25° in LD cycles using DAM2 monitors (Trikinetics) and sleep was measured beginning 36–48 hr after animals were transferred to tubes, to permit acclimation to tubes and to allow feeding for conditional induction experiments. Locomotor data were collected in 1 min bins, and inactivity greater than 5 min (Shaw *et al.* 2000; Huber *et al.* 2004) was used to define sleep. Sleep parameters were analyzed with custom MATLAB (Mathworks) software (Stavropoulos and Young 2011). Dead animals were excluded from analysis by a combination of automated filtering and visual inspection of locomotor traces.

### Statistical analysis

For analysis of RU486 toxicity, one-way ANOVA and Tukey-Kramer *post hoc* tests were used to compare within each genotype. For statistical analysis of total sleep, daytime sleep, and nighttime sleep, one-way ANOVA and Tukey–Kramer *post hoc* tests were used to compare between induction conditions for each genotype. For comparisons of sleep bout length, nonparametric Kruskal–Wallis tests followed by Dunn’s *post hoc* tests were used.

### Immunohistochemistry

For preparation of larval brains, crosses of *tub-QS*; *nsyb-Gal4QF* and *UAS-CD8-GFP* animals were set on food containing quinic acid. Brains of third instar larvae were dissected in PBS, fixed with 4% paraformaldehyde in PBS for 30 min at room temperature, and washed 3 × 15 min at room temperature in PBS containing 0.2% Triton X-100 (PBST). For preparation of adult brains, crosses of *tub-QS*; *nsyb-Gal4QF* and *UAS-CD8-GFP* animals were set on standard fly food. One- to four-day-old young adult males eclosing from these crosses were moved to food containing quinic acid for 1 wk, fixed with 4% paraformaldehyde in PBST at 4° for 3 hr, and washed 3 × 15 min in PBST at room temperature before brain dissection. Dissected larval and adult brains were blocked with 5% normal donkey serum (NDS) (Lampire Biological, 7332100) in PBST at room temperature for 30 min, and incubated in primary antibody cocktail overnight at 4°, followed by 3 × 15 min PBST washes at room temperature. Brains were subsequently incubated in secondary antibody cocktail overnight at 4°, washed 3 × 15 min at room temperature in PBST, and mounted on microscope slides in Vectashield (Vector Labs, H-1000). Antibody cocktails were prepared in 5% NDS in PBST. Primary antibodies were rabbit anti-GFP (Life Technologies, A11122) used at 1:500, and mouse nc82 anti-Bruchpilot (DSHB) used at 1:50. Secondary antibodies were Alexa 488 donkey anti-rabbit and Alexa 647 donkey anti-mouse (Life Technologies, A21206 and A31573), both used at 1:1000. Brains were imaged on a Zeiss LSM800 confocal microscope at 512 × 512 or 1024 × 1024 pixel resolution with 0.95 μM z-steps. Confocal images were processed in ImageJ by collapsing z-stacks into single images using maximum intensity projection.

### Data availability

Fly stocks and locomotor data from this study are available upon request. The authors state that all data necessary for confirming the conclusions presented in the article are represented fully within the article.

## Results

### Constitutive, developmental-specific, and adult-specific neuronal induction using Geneswitch and the Q-system

**Figure 1:**
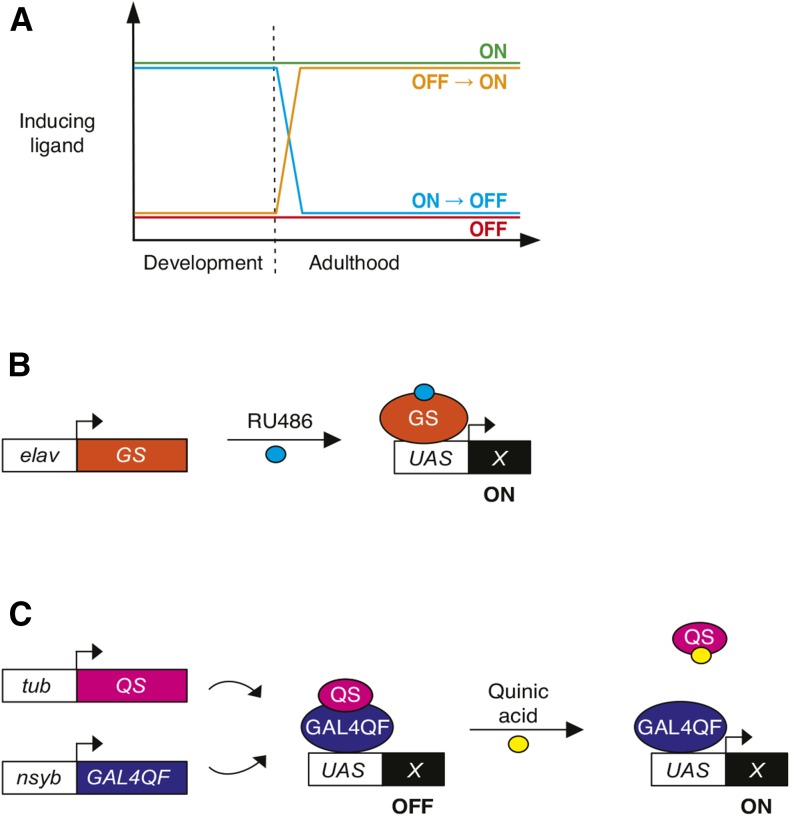
Conditional neuronal manipulations using ligand-inducible systems. (A) Schematic depicting regimens for vehicle control (**OFF**), and constitutive (**ON**), developmental-specific (**ON→OFF**), and adult-specific (**OFF→ON**) induction by temporally restricted delivery of inducing ligands. See Materials and Methods for additional details. (B) The *elav-GS* transgene encodes a neuronally expressed, RU486-inducible form of the Gal4 activator, which activates a UAS-driven effector transgene (X) in the presence of RU486. (C) The Q-system is composed of the chimeric GAL4QF activator, the quinic acid-sensitive QS suppressor, and a UAS-driven effector transgene (X). Ubiquitous and constitutive *tub-QS* expression suppresses neuronally expressed GAL4QF. Quinic acid derepresses the UAS transgene.

To determine whether Geneswitch and the Q-system can be used to assess developmental and adult contributions of genes relevant to sleep, we tested these systems in four different regimens of ligand exposure (Figure 1A). The nomenclature of these regimens indicates the presence or absence of inducing ligand during development and subsequently during adulthood and behavioral assay. In the noninduced control condition (**OFF**), animals developed on vehicle-containing food and were maintained in the absence of ligand throughout adulthood and behavioral assay. For adult-specific induction (**OFF→ON**), animals developed in the presence of vehicle but were moved as young adults to ligand-containing food for behavioral assay. For developmental-specific induction (**ON→OFF**), ligand was present during development and young adults were moved to vehicle-containing food for behavioral assay. Finally, for constitutive induction (**ON**), ligand was present throughout development, adulthood, and behavioral assay. Because many genes implicated in regulating sleep function within neurons, we used panneuronally expressed drivers for our conditional manipulations: *elav-GS* for the Geneswitch system (Figure 1B), and *nsyb-Gal4QF* for the Q-system (Figure 1C).

### Early RU486 exposure causes developmental defects in flies bearing elav-GS

**Figure 2:**
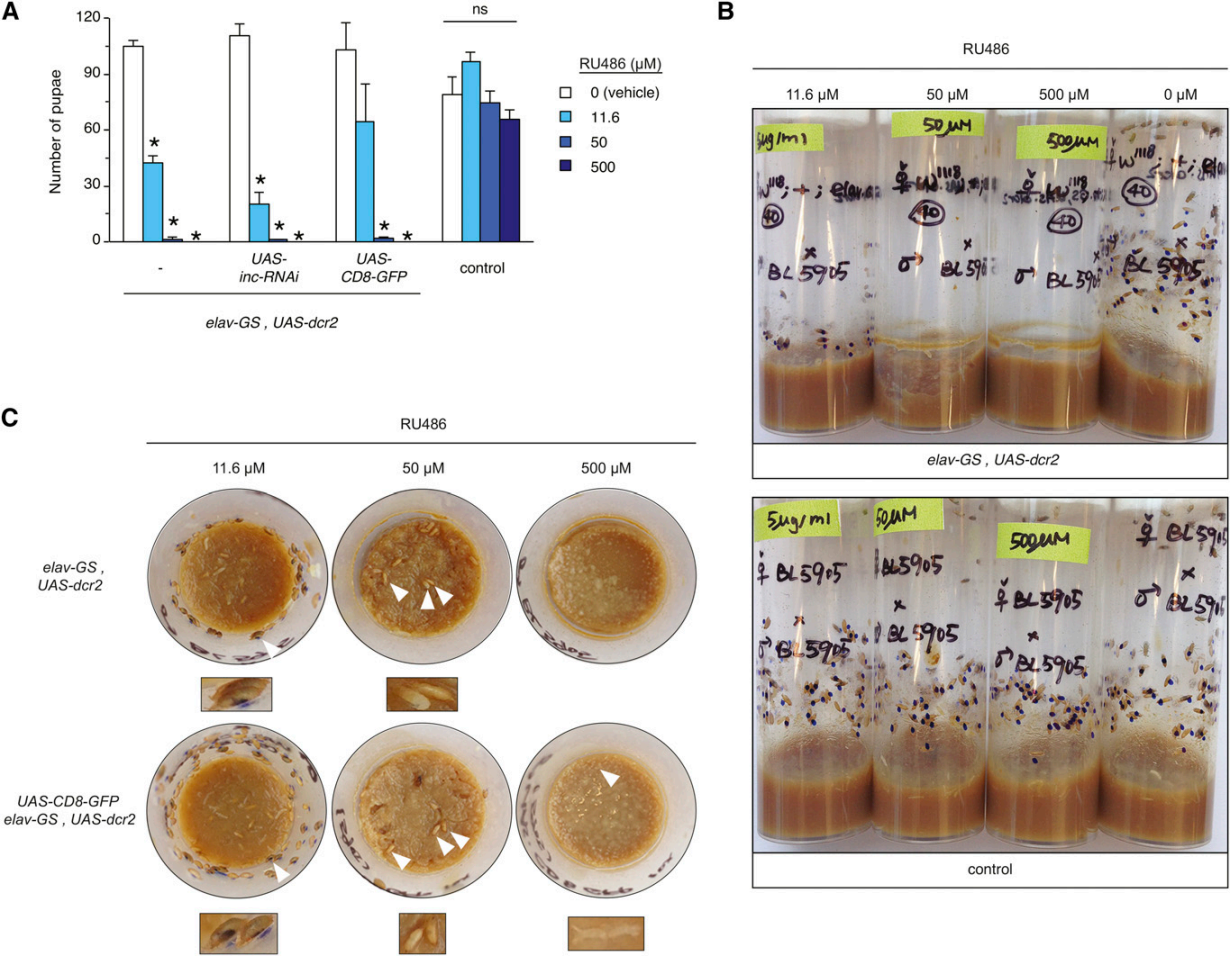
Developmental RU486 exposure is toxic to *elav-GS* animals. (A) Pupal number is shown for indicated genotypes exposed developmentally to vehicle or to indicated RU486 concentrations. Mean ± SEM is shown; * *P* < 0.01, and ns denotes *P* > 0.05, for comparisons to vehicle control within each genotype. Data are averaged from two independently derived *elav-GS*, *UAS-dcr2* recombinant lines. (B and C) Side view (B) and top-down (C) photographs of vials containing progeny of indicated genotypes. Photographs were taken 11 d after crosses were initiated. Pupal cases are marked with blue dots to facilitate counting and to distinguish them from adults that have eclosed. In (C), white arrowheads indicate mature pigmented pupae on vial walls (11.6 µM RU486), immature unpigmented pupae located on the food surface (50 µM), and undeveloped eggs and embryos (500 µM). Magnifications are shown underneath top-down photographs.

Conditional loss-of-function enables the temporal requirements of genes to be assessed with respect to sleep and can be achieved by driving RNAi with the Geneswitch system. We therefore first assessed animals which carry *elav-GS* and the *UAS-dcr2* transgene expressing the Dcr2 ribonuclease that enhances RNAi (Dietzl *et al.* 2007), both of which are used routinely for studies of sleep (*e.g.*, Yuan *et al.* 2006; Stavropoulos and Young 2011; Pfeiffenberger and Allada 2012; Ueno *et al.* 2012). To test whether continuous *elav-GS* induction is compatible with development and with behavioral assay of sleep, we set crosses on RU486 concentrations (11.6, 50, and 500 μM) that span a ∼50-fold range and encompass concentrations typically used in studies of sleep. While we observed similar numbers of pupae for *elav-GS*, *UAS-dcr2/+* and isogenic *w^1118^* control animals exposed developmentally to ethanol vehicle, the presence of RU486 had sharply contrasting effects on the two genotypes (Figure 2A). The number of control pupae was not significantly reduced in the presence of RU486 (Figure 2, A and B), indicating that these animals tolerate high levels of RU486 through pupal development. In contrast, the numbers of pupae bearing *elav-GS* were reduced in a dose-dependent manner when RU486 was present throughout development (Figure 2, A and B). Pupae bearing *elav-GS* failed to develop at 500 μM RU486, with abundant eggs or embryos and a lack of larvae suggesting lethality at early developmental stages (Figure 2, A–C). At 50 μM RU486, few *elav-GS*-bearing larvae and pupae were observed, and at 11.6 μM RU486, the number of pupae was less than half of that observed for vehicle controls (Figure 2, A–C). We observed similar developmental toxicity in *elav-GS*, *UAS-dcr2/+* animals that also carried *UAS-inc-RNAi* or *UAS-CD8-GFP* transgenes, with >98% reductions in numbers of pupae for both genotypes at 500 and 50 μM RU486, and 82 and 37% reductions respectively at 11.6 μM RU486 (Figure 2, A and C). Similar developmental toxicity was observed when *elav-GS* was inherited maternally (Figure 2) or paternally (Supplemental Material, Figure S1), indicating that these effects are unlikely to reflect parent-specific contributions or reproductive deficits, and are instead likely to arise in progeny bearing the *elav-GS* driver.

In addition to reduced brood size, we observed additional developmental defects that were dependent on the presence of *elav-GS* and the dose of RU486. Experimental genotypes exposed to 50 µM RU486 exhibited developmental delays, as indicated by a lack of pupal pigmentation in comparison to animals exposed to 11.6 µM RU486, or to control animals scored at the same time point (Figure 2, B and C). Furthermore, *elav-GS*-bearing animals exposed to 50 µM RU486 pupated primarily on the surface of food rather than on vial walls (Figure 2, B and C). Control animals lacking *elav-GS* pupated normally when exposed developmentally to RU486 (Figure 2, A and B). Taken together, these findings strongly suggest that developmental *elav-GS* activation is toxic and that compromised neuronal function may underlie this toxicity.

### Developmental induction of elav-GS causes strong and nonspecific alterations of sleep

While developmental exposure to moderate and high concentrations of RU486 blocked development to adulthood, the lowest concentration we tested (11.6 μM) permitted eclosion of animals bearing *elav-GS* and additional UAS transgenes (Figure 2, A–C). We therefore used this concentration for developmental-specific induction (**ON→OFF**), and for the developmental portion of the constitutive induction (**ON**) regimen (Figure 1A); adult animals in the latter regimen were fed higher RU486 concentrations (500 µM) after eclosion as in prior studies. We assessed the four induction conditions in animals carrying *elav-GS*, *UAS-dcr2*, and an RNAi transgene directed against *insomniac* (*inc*), a gene we previously isolated in a chemical mutagenesis screen for short sleep mutants (Stavropoulos and Young 2011). *inc* null mutants exhibit severely curtailed sleep, and this phenotype is recapitulated in animals in which *inc* RNAi is driven by *elav-Gal4*, indicating that *inc* is required neuronally (Stavropoulos and Young 2011; Pfeiffenberger and Allada 2012).

**Figure 3:**
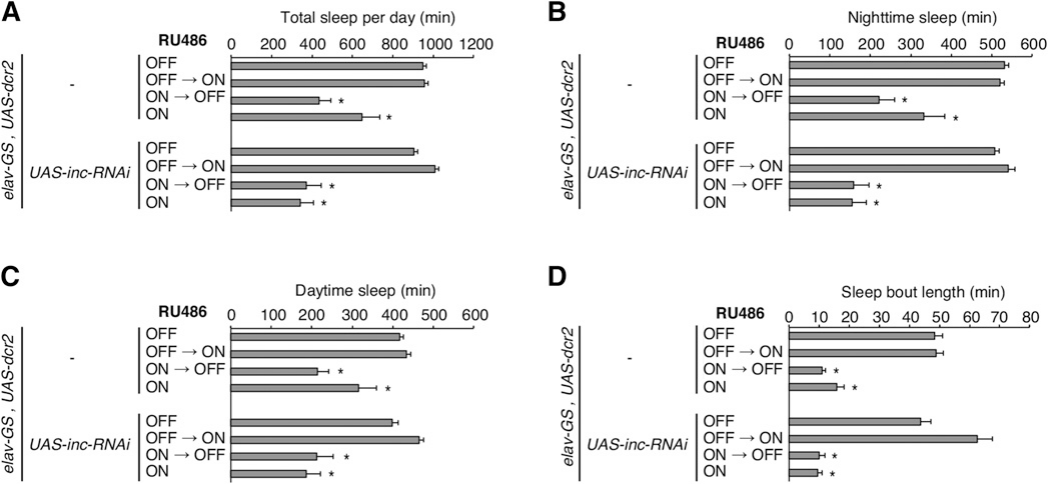
Developmental or continuous RU486 exposure reduces sleep in *elav-GS* adults. (A–D) Sleep parameters are shown for animals of indicated genotypes and regimens of RU486 exposure. Total sleep per day (A), nighttime sleep (B), daytime sleep (C), and sleep bout length (D) are plotted. Mean ± SEM is shown; *n* = 18–38; * *P* < 0.01 for comparisons to vehicle control condition within each genotype.

Animals bearing *elav-GS*, *UAS-dcr2*, and *UAS-inc-RNAi* exhibited a severe reduction of sleep when they were exposed to RU486 specifically during development (**ON→OFF**) or constitutively (**ON**); no change in sleep was observed when RU486 was absent (**OFF**) or fed to adults (**OFF→ON**) (Figure 3A). These results are consistent with earlier findings, in which reduced sleep was reported for developmental-specific induction of *inc* RNAi using the same *elav-GS* driver (Pfeiffenberger and Allada 2012). Surprisingly, however, control animals lacking *UAS-inc-RNAi* showed similarly decreased sleep when these animals were exposed to RU486 developmentally (**ON→OFF**) or constitutively (**ON**), but not when RU486 was absent (**OFF**) or present only in adulthood (**OFF→ON**) (Figure 3A). In addition to exhibiting strong reductions in total sleep, both genotypes displayed qualitatively and quantitatively similar decreases in daytime sleep, nighttime sleep, and in sleep bout length after developmental (**ON→OFF**) or constitutive (**ON**) RU486 exposure (Figure 3, B–D). These results indicate that the reductions in sleep observed in animals carrying *UAS-inc-RNAi*, *elav-GS*, and *UAS-dcr2* are unlikely to arise from the depletion of *inc*, but rather, from nonspecific alterations of sleep that are similarly observed in control animals lacking the *UAS-inc-RNAi* transgene.

**Figure 4:**
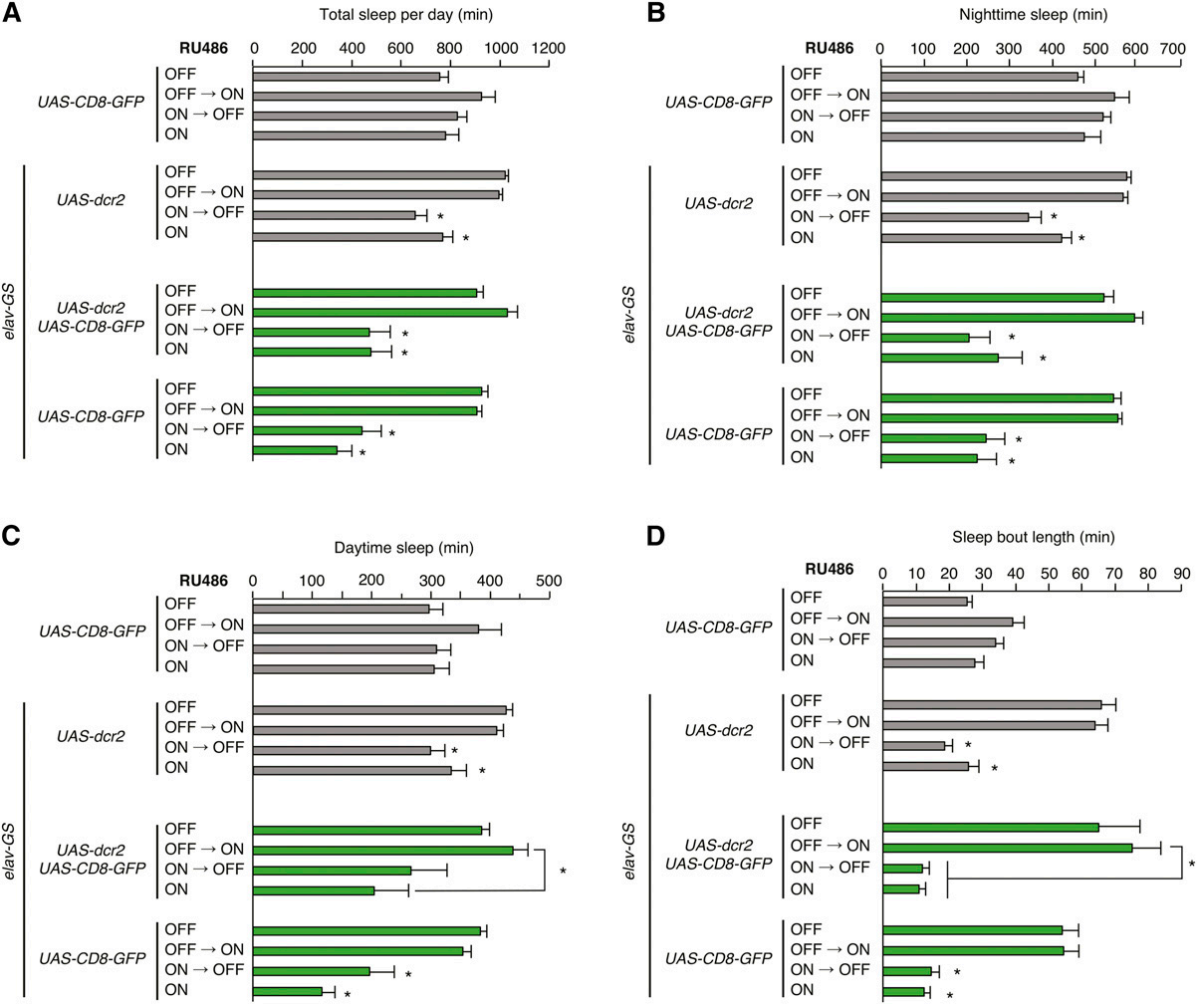
Perturbations of sleep elicited by developmental RU486 exposure are dependent on *elav-GS* and independent of effector transgenes. (A–D) Sleep parameters are shown for animals of indicated genotypes and regimens of RU486 exposure. Total sleep per day (A), nighttime sleep (B), daytime sleep (C), and sleep bout length (D) are plotted. Mean ± SEM is shown; *n* = 6–40; * *P* < 0.01 for comparisons to vehicle control condition within each genotype except where indicated.

To further test whether *elav-GS* elicits nonspecific alterations in sleep when induced developmentally or constitutively, we assessed sleep in animals bearing *elav-GS*, *UAS-dcr2*, and *UAS-CD8-GFP*, a transgene unrelated to sleep. Surprisingly, we observed strong reductions in sleep when these animals were exposed to RU486 developmentally (**ON→OFF**) or constitutively (**ON**); animals exposed to vehicle (**OFF**) or to RU486 specifically in adulthood (**OFF→ON**) exhibited wild-type levels of sleep (Figure 4A). We performed additional experiments to assess whether reductions of sleep were dependent on the *elav-GS*, *UAS-dcr2*, and *UAS-CD8-GFP* transgenes. Perturbations of sleep were similarly severe in animals carrying only *elav-GS* and *UAS-CD8-GFP*, indicating that these effects do not depend on the presence of *UAS-dcr2* (Figure 4A). Animals bearing only *UAS-CD8-GFP* showed no significant alterations of sleep in all four regimens of RU486 exposure, indicating that reductions in sleep require *elav-GS* (Figure 4A). All genotypes with decreased sleep exhibited strongly reduced sleep during the daytime and at night, as well as reduced sleep bout length (Figure 4, B–D). These findings indicate that developmental induction of *elav-GS* strongly and nonspecifically perturbs sleep. As was the case for developmental toxicity (Figure 2 and Figure S1), altered sleep in *elav-GS* animals exposed developmentally to RU486 was observed regardless of whether the *elav-GS* transgene was inherited maternally or paternally (Figure 4 and Figure S2). These data strongly suggest that sleep defects are caused by altered nervous system function as a consequence of developmental *elav-GS* activation.

In further experiments, we extended our analysis to additional genotypes carrying *elav-GS* and *UAS-cDNA* or *UAS-RNAi* transgenes. In total, we assayed ∼1800 animals representing 19 different genotypes in the four induction regimens described above. For all of these genotypes, we consistently observed strongly reduced sleep when animals bearing *elav-GS* were exposed to RU486 developmentally or constitutively (data not shown). These results strongly support the conclusion that these reductions of sleep are independent of particular effector transgenes and genetic background, and that they occur instead from developmental *elav-GS* activation. While our findings confirm that *elav-GS* and RU486 are well tolerated in adults and are compatible with adult-specific conditional manipulations of sleep, they indicate that developmental induction of *elav-GS*, even at low concentrations of RU486, alters sleep acutely and nonspecifically during adulthood, confounding the use of this driver for developmental manipulations of sleep.

### Neuronal induction of the Q-system is broadly compatible with assessing sleep

We next sought to assess the suitability of the Q-system for conditional neuronal manipulations of sleep. For these experiments we used the panneuronally expressed Gal4QF activator (*nsyb-Gal4QF*), the ubiquitously expressed tubulin-QS suppressor (*tub-QS*), and quinic acid (Figure 1C). While quinic acid has no obvious toxicity in *Drosophila* (Potter *et al.* 2010), its effects on sleep have not been assessed. Similarly, animals bearing *QF* and *QS* transgenes have not been tested in the presence and absence of quinic acid to assess compatibility with behavioral assays of sleep.

**Figure 5:**
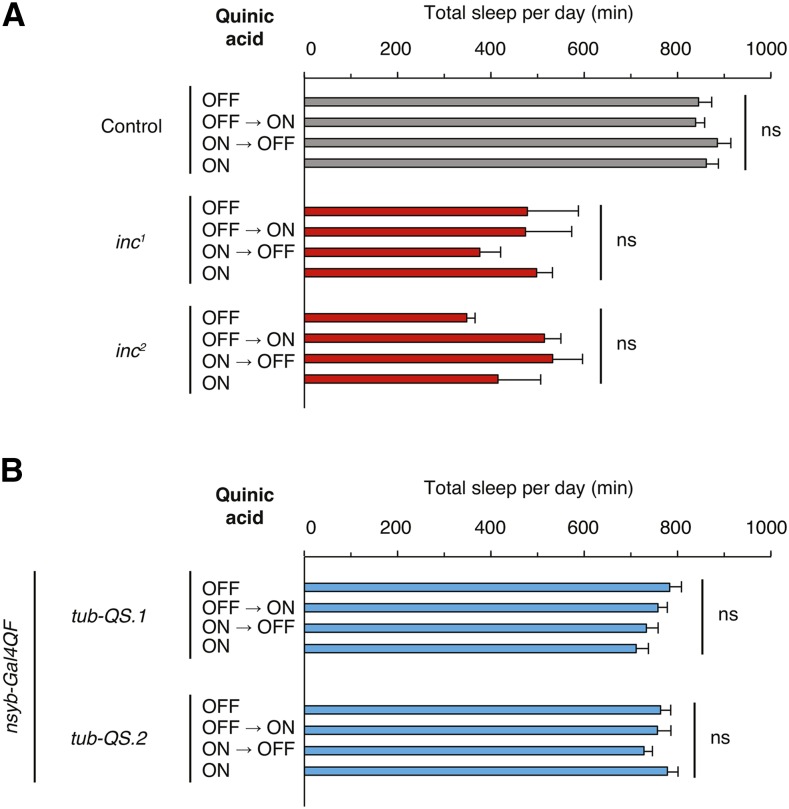
The Q-system is compatible with assessing sleep. (A and B) Total sleep per day is plotted for animals of indicated genotypes and regimens of quinic acid exposure. Mean ± SEM is shown; *n* = 5–16 for (A); *n* = 22–24 for (B); ns denotes *P* > 0.05, for comparisons across induction conditions within each genotype.

First, we assessed whether quinic acid exposure alters sleep in control animals lacking Q-system transgenes. In these animals, quinic acid exposure during development, adulthood, or both did not alter sleep with respect to vehicle control (Figure 5A). We next assessed whether quinic acid exposure alters the sleep of *inc^1^* and *inc^2^* animals, which bear null alleles of *insomniac* that strongly curtail sleep (Stavropoulos and Young 2011). Quinic acid supplied developmentally or during adulthood did not significantly change the short sleep phenotype of either mutant, indicating that quinic acid exposure does not perturb the levels of sleep in a short-sleeping genetic background (Figure 5A).

Next, we tested animals carrying the *nsyb-Gal4QF* and *tub-QS* transgenes necessary for conditional, neuronally restricted Q-system manipulations. The presence of quinic acid during development, adulthood, or both, did not significantly alter sleep with respect to vehicle control (Figure 5B and Figure S3), and this was the case for two stocks bearing different insertions of the *tub-QS* transgene. These findings contrast with those obtained with neuronally expressed Geneswitch (Figure 3 and Figure 4), and indicate that persistent quinic acid exposure is well tolerated and does not alter sleep in the presence of Q-system components (Figure 5, A and B). Furthermore, quinic acid exposure does not cause any obvious developmental abnormalities (data not shown).

**Figure 6:**
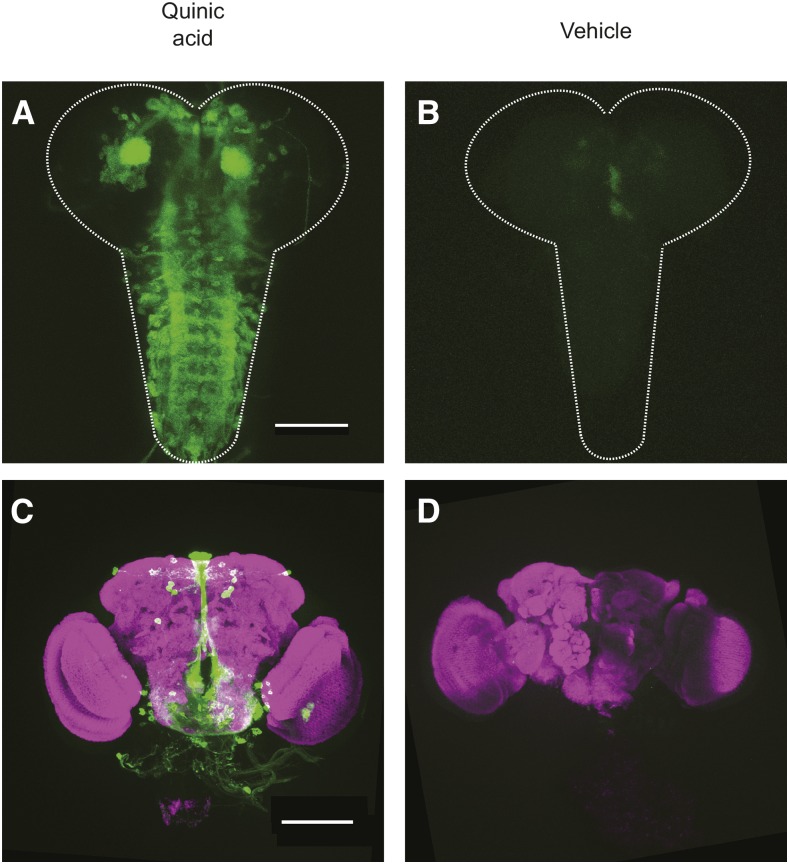
Conditional neuronal induction of a GFP reporter with the Q-system. (A and B) Anti-GFP signal (green) in third instar larval brains prepared from *tub-QS*/+; *nsyb-Gal4QF*/*UAS-CD8-GFP* animals exposed developmentally to (A) quinic acid or (B) vehicle. (C and D) anti-GFP (green) and anti-Bruchpilot (magenta) signal in adult brains prepared from *tub-QS*/+; *nsyb-Gal4QF*/*UAS-CD8-GFP* animals exposed during adulthood to (C) quinic acid or (D) vehicle. Scale bars represent 100 µm.

To test whether conditions compatible with assessing sleep enable conditional induction of transgenes with the Q-system, we assessed expression of a *UAS-CD8-GFP* reporter in animals bearing *nsyb-Gal4QF* and *tub-QS* and exposed to quinic acid developmentally or during adulthood. Developmental exposure to quinic acid elicited *UAS-CD8-GFP* expression, as assessed in the brains of third instar larvae (Figure 6A). Animals developing in the presence of vehicle exhibited little to no GFP signal (Figure 6B). Similarly, the brains of adult animals exposed to quinic acid in adulthood exhibited GFP signal (Figure 6C) similar to that reported previously (Riabinina *et al.* 2015), while vehicle controls exhibited no signal (Figure 6D). While the kinetics and efficacy of Q-system induction are likely to vary with each effector transgene, the conditional induction of GFP expression and the compatibility of inducing conditions with behavioral analysis of sleep (Figure 5) suggest that the Q-system has considerable potential for dissecting the temporal contributions of genes that impact sleep.

## Discussion

Conditional genetic manipulations provide important information for defining the temporal window in which genes exert their phenotypic consequences. For genes implicated in regulating sleep, temporal requirements vis-à-vis sleep have been assessed chiefly in an adult-specific manner using the Geneswitch system. While our results confirm that the panneuronally expressed *elav-GS* transgene is compatible with adult-specific manipulations as reported previously (Yuan *et al.* 2006; Joiner *et al.* 2006; Seugnet *et al.* 2008, 2011; Bushey *et al.* 2009; Donlea *et al.* 2009; Wu *et al.* 2009; Kuo *et al.* 2010, 2012; Crocker *et al.* 2010; Ishimoto and Kitamoto 2010; Pfeiffenberger and Allada 2012; Erion *et al.* 2012; Ueno *et al.* 2012; Vanderheyden *et al.* 2013; Tulina *et al.* 2014; Liu *et al.* 2014; Kayser *et al.* 2014; Oh *et al.* 2014; Chen *et al.* 2015; Tabuchi *et al.* 2015; Dissel *et al.* 2015; Afonso *et al.* 2015), they indicate that developmental *elav-GS* induction elicits strong, nonspecific alterations of sleep later in adulthood. Notably, these sleep deficits occur in animals exposed to low concentrations (11.6 µM) of RU486, nearly 50-fold below those typically used for adult manipulations (500 µM). Transcriptome profiling studies have identified genes whose expression is altered by RU486 exposure (Etter *et al.* 2005; Landis *et al.* 2015), but whether these changes underlie *elav-GS*-dependent alterations of sleep or mediate developmental toxicity awaits further investigation.

While many studies of sleep have employed adult-specific *elav-GS* induction, few have reported developmental manipulations, despite the obvious value of such manipulations for assessing the contributions that genes may make outside of adulthood. We suspect that this relative dearth of studies reflects complications of developmental induction that have been observed but not widely reported. This notion is supported by both published and unpublished findings that suggest a threshold for developmental toxicity of RU486 in *elav-GS* animals similar to what we have observed. In one study, developmental lethality was reported above 25 µM RU486 for animals bearing *elav-GS* and effector transgenes (van Swinderen 2007). In unpublished findings that parallel our results, concentrations above 50 µM RU486 were found to be developmentally lethal to *elav-GS* animals; at 50 µM RU486, some animals were observed to eclose, but viability was reduced (W. Joiner, personal communication). In other unpublished studies, developmental lethality and alterations of sleep in some genotypes bearing *elav-GS* were observed with developmental exposure to 25 µM RU486 (J. Williams, personal communication). Though our results suggest that developmental toxicity in *elav-GS* animals is significantly reduced at 11.6 µM RU486, a concentration permissive for the development of animals carrying ∼200 different larvally expressed Geneswitch transgenes (Nicholson *et al.* 2008), nonspecific alterations of sleep persist at this concentration across a wide range of genotypes.

Our findings suggest that developmental manipulations using *elav-GS* need to be interpreted cautiously in the context of assessing sleep, and that some earlier results may need to be revisited. While our findings do not preclude developmental functions of *inc* and *Cul3* with respect to sleep, the interpretational difficulties associated with developmental *elav-GS* induction suggest that conditional systems other than Geneswitch may be required to resolve the temporal requirements of *inc* and *Cul3* conclusively. Our results emphasize the necessity of validating the kinetics and efficacy of conditional manipulations in parallel to behavioral assays, in order to interpret phenotypes of these manipulations. This point is underscored by the finding that Geneswitch activation depends upon the age and sex of animals, and that some Geneswitch drivers are expressed in unanticipated locations or in the absence of RU486 inducer (Poirier *et al.* 2008). Finally, we note that sleep is a behavior sensitive to a number of factors, and that genetic background, food composition, or environmental conditions may play a role in the developmental toxicity and nonspecific alterations of sleep that result from *elav-GS* activation during development.

More broadly, our findings are relevant for conditional neuronal manipulations of other behaviors using ligand-inducible expression systems. Developmental activation of *elav-GS* may cause persistent changes in the structure or activity of the nervous system and perturb other behaviors nonspecifically, potentially limiting the use of this driver to adulthood. While the Q-system requires validation in additional behavioral contexts, our findings suggest that it may have broader potential for conditional neuronal manipulations throughout the *Drosophila* life cycle and for systematically defining the temporal contributions of genes that underlie various behaviors.

## Supplementary Material

Supplemental Material
