# Supplementary material for: Evaluation of Ligand-Inducible Expression Systems for Conditional Neuronal Manipulations of Sleep in Drosophila
Source: G3 (Bethesda). 2016 Aug 23;6(10):3351–9. doi: 10.1534/g3.116.034132 (PMC5068954; doi:10.1534/g3.116.034132)
Supplement: Supplemental Material [file supp_6_10_3351__index.html]

Evaluation of Ligand-Inducible Expression Systems for Conditional Neuronal Manipulations of Sleep in Drosophila — Supplemental Material 

# Evaluation of Ligand-Inducible Expression Systems for Conditional Neuronal Manipulations of Sleep in *Drosophila*

## Supplemental Material for Li and Stavropoulos, 2016

**Files in this Data Supplement:**

- Figure S1 - Developmental RU486 exposure is toxic to animals inheriting *elav-GS* paternally (.pdf, 2,128 KB)
- Figure S2 - Developmental or continuous RU486 exposure reduces sleep in animals inheriting *elav-GS* paternally. (.pdf, 963 KB)
- Figure S3 - Additional sleep parameters for animals undergoing neuronal Q-system induction. (.pdf, 945 KB)
