# Supplementary material for: Evaluation of Ligand-Inducible Expression Systems for Conditional Neuronal Manipulations of Sleep in Drosophila
Source: G3 (Bethesda). 2016 Aug 23;6(10):3351–9. doi: 10.1534/g3.116.034132 (PMC5068954; doi:10.1534/g3.116.034132)
Supplement: Supplemental Material [file supp_g3.116.034132_FigureS1.pdf]

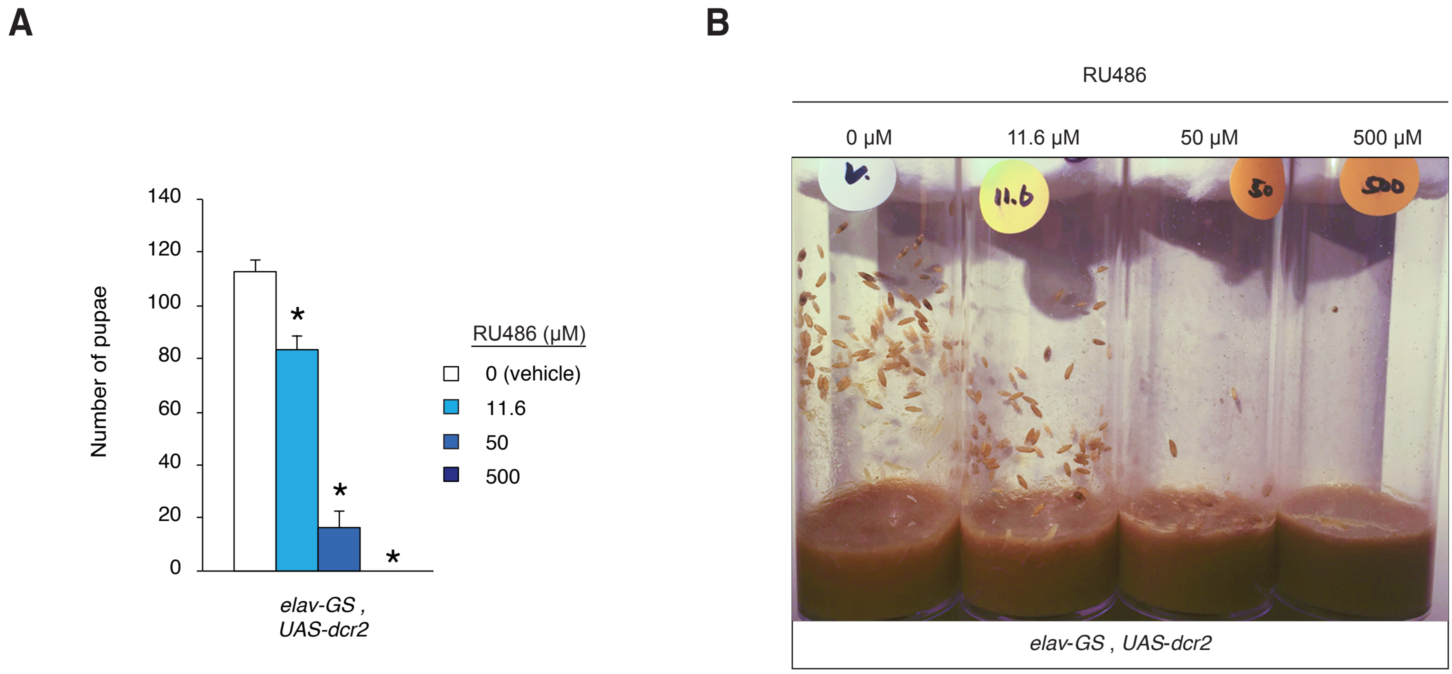


**Figure S1**

**Developmental RU486 exposure is toxic to animals inheriting *elav-GS* paternally**

**A)** Pupal number is shown for animals inheriting indicated transgenes paternally and exposed developmentally to vehicle or to indicated RU486 concentrations. Mean ± SEM is shown; * $p$<0.01, for comparisons to vehicle control. Data are averaged from two independently derived *elav-GS* , *UAS-dcr2* recombinant lines.

**B)** Side view photographs of vials containing progeny bearing indicated transgenes inherited paternally. Photographs were taken 10 days after crosses were initiated.
