# Supplementary material for: Evaluation of Ligand-Inducible Expression Systems for Conditional Neuronal Manipulations of Sleep in Drosophila
Source: G3 (Bethesda). 2016 Aug 23;6(10):3351–9. doi: 10.1534/g3.116.034132 (PMC5068954; doi:10.1534/g3.116.034132)
Supplement: Supplemental Material [file supp_g3.116.034132_FigureS2.pdf]

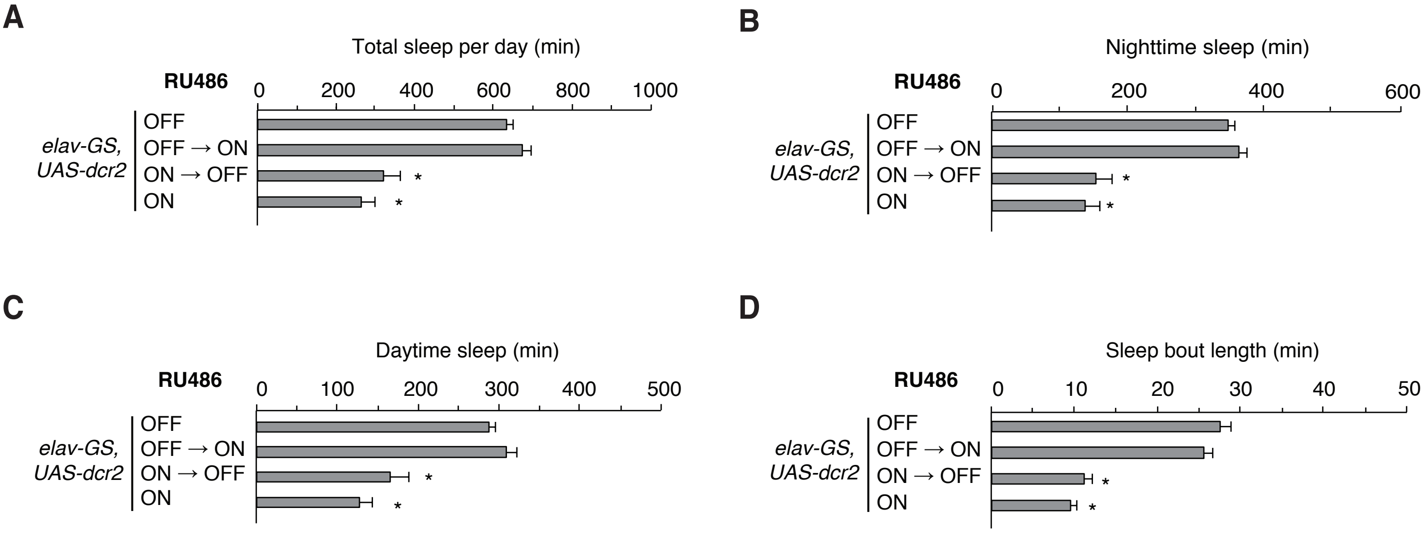

Figure S2

Developmental or continuous RU486 exposure reduces sleep in animals inheriting *elav-GS* paternally

(A-D) Sleep parameters are shown for animals inheriting the *elav-GS* and *UAS-dcr2* transgenes paternally and exposed to RU486 as indicated. Total sleep per day **(A)**, nighttime sleep **(B)**, daytime sleep **(C)**, and sleep bout length **(D)** are plotted. Mean ± SEM is shown; n = 25-32, * p<0.01 for comparisons to vehicle control condition within each genotype.
