# Supplementary material for: Evaluation of Ligand-Inducible Expression Systems for Conditional Neuronal Manipulations of Sleep in Drosophila
Source: G3 (Bethesda). 2016 Aug 23;6(10):3351–9. doi: 10.1534/g3.116.034132 (PMC5068954; doi:10.1534/g3.116.034132)
Supplement: Supplemental Material [file supp_g3.116.034132_FigureS3.pdf]

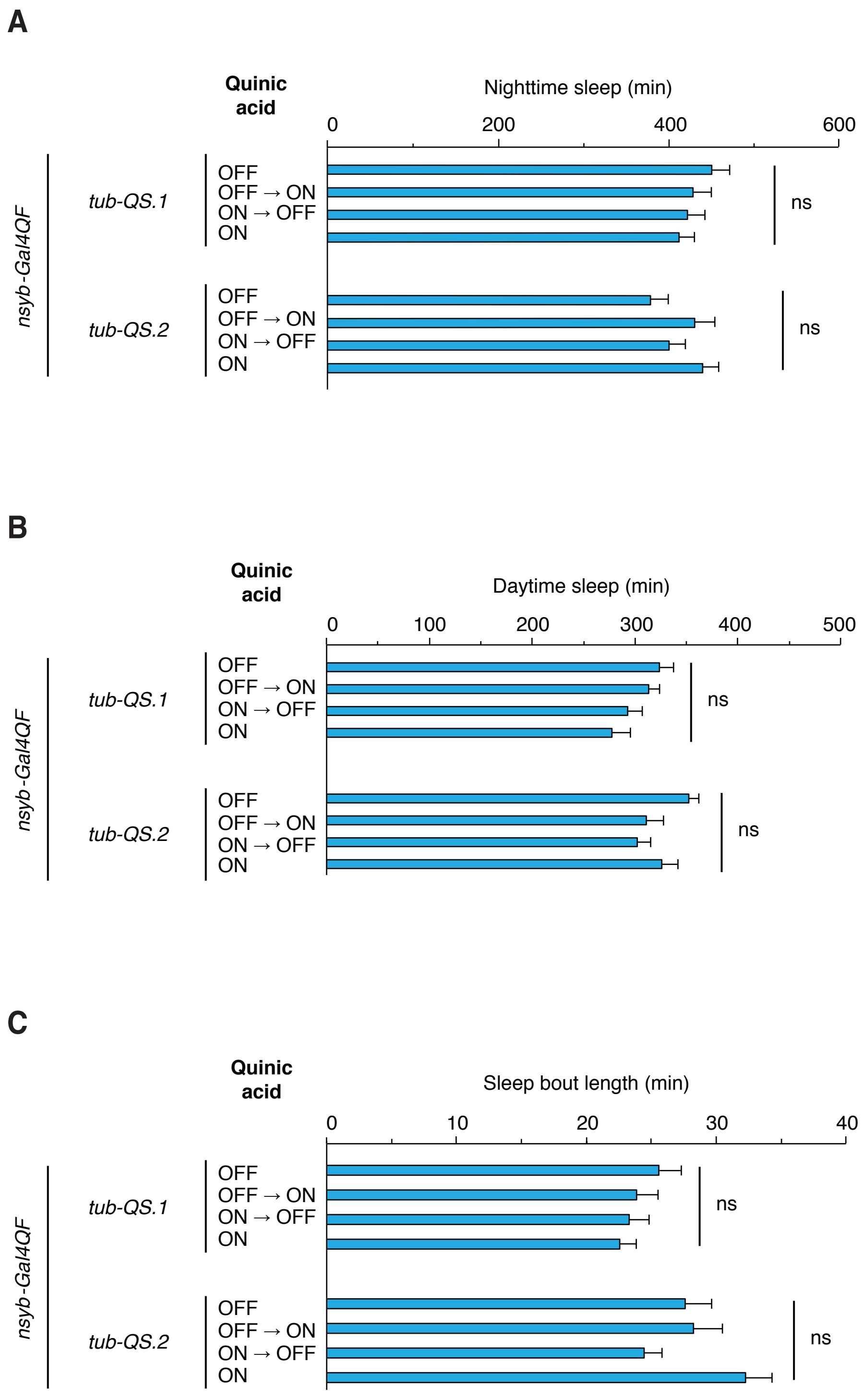

Figure S3

Additional sleep parameters for animals undergoing neuronal Q-system induction

(A-C) Average **(A)** nighttime sleep, **(B)** daytime sleep, and **(C)** sleep bout length is shown for animals bearing *tub-QS* and *nsyb-GAL4QF* and exposed to quinic acid as indicated. Mean ± SEM is shown; n = 22-24, ns indicates $p>0.05$.
